# Manual Therapy With Rest as a Treatment for Established Inflammation and Fibrosis in a Rat Model of Repetitive Strain Injury

**DOI:** 10.3389/fphys.2021.755923

**Published:** 2021-11-04

**Authors:** Mary F. Barbe, Siva Tejaa Panibatla, Michele Y. Harris, Mamta Amin, Jocelynne T. Dorotan, Geneva E. Cruz, Geoffrey M. Bove

**Affiliations:** ^1^Center for Translational Medicine, Lewis Katz School of Medicine, Temple University, Philadelphia, PA, United States; ^2^Bove Consulting, Kennebunkport, ME, United States

**Keywords:** overuse injury, work-related musculoskeletal disorders, massage therapy, repetitive motion disorder, fibrosis, neuropathy

## Abstract

**Background**: Repetitive strain injuries caused by repetitive occupational work are difficult to prevent for multiple reasons. Therefore, we examined the effectiveness of manual therapy (MT) with rest to treat the inflammation and fibrosis that develops through the performance of a repetitive task. We hypothesized that this treatment would reduce task-induced sensorimotor declines and neuromuscular inflammation.

**Methods**: Twenty-nine female Sprague-Dawley rats performed a reaching and lever-pulling task for 14weeks. All ceased performing the task at 14weeks. Ten were euthanized at this timepoint (TASK). Nine received manual therapy to their upper extremities while resting 7weeks (MTR); 10 were assigned to rest alone (REST). Ten additional food restricted rats were included that neither performed the task nor received manual therapy (FRC).

**Results**: Confirming previous experiments, TASK rats showed behavioral changes (forepaw mechanical hypersensitivity, reduced grip strength, lowered forelimb/forepaw agility, and noxious cold temperature sensitivity), reduced median nerve conduction velocity (NCV), and pathological tissue changes (myelin degradation, increased median nerve and muscle inflammation, and collagen production). Manual therapy with rest (MTR) ameliorated cold sensitivity seen in REST rats, enhanced muscle interleukin 10 (IL-10) more than in REST rats, lead to improvement in most other measures, compared to TASK rats. REST rats showed improved grip strength, lowered nerve inflammation and degraded myelin, and lowered muscle tumor necrosis factor alpha (TNFα) and collagen I levels, compared to TASK rats, yet maintained lowered forelimb/forepaw agility and NCV, and increased neural fibrosis.

**Conclusion**: In our model of repetitive motion disorder, manual therapy during rest had modest effects on behavioral, histological, and physiological measures, compared to rest alone. These findings stand in contrast to the robust preventive effects of manual therapy in this same model.

## Introduction

Musculoskeletal disorders secondary to repetitive occupational overuse are highly prevalent in many professions and include repetitive strain injuries (Panagos et al., 2007; Wang et al., 2009; Panush, 2017; Epstein et al., 2018). For example, in 2018, there were 900,380days away from work cases in the United States private sector with 30 percent of the cases being due to musculoskeletal disorders (Bureau of Labor Statistics, 2020). Repetitive strain injury is caused by repetitive, sustained, and forceful movements (Byl and McKenzie, 2000; Gallagher and Heberger, 2013). These injuries and disorders have profound effects on the neuromuscular system (which includes nerves and muscles; NIOSH, 1997). Repeated injury is associated with chronic inflammation and fibrosis (erratically formed collagen or scar) in tissues undergoing chronic injury/repair processes (Barr and Barbe, 2002; Kjaer, 2004).

We have a rat model of occupational repetitive strain injury (also considered an overuse injury model), in which rats voluntarily reach and pull on a lever bar at target reach rates and force levels for a food reward (Barbe et al., 2013). In this model, operant reach rates and force levels were determined from studies of risk exposure of humans performing similar work tasks (Silverstein et al., 1986; Barr and Barbe, 2002). We have shown that prolonged performance of a relatively high repetition and force lever-pulling task induces sensorimotor declines, and broad neuromuscular inflammation and fibrosis (Clark et al., 2004; Fedorczyk et al., 2010; Abdelmagid et al., 2012; Barbe et al., 2013, 2020a; Fisher et al., 2015; Bove et al., 2016, 2019).

Manual therapy (MT) is any therapy that is applied to using the hands, including massage therapy and manipulative therapy. Manual therapy has been practiced for centuries primarily for musculoskeletal disorders, repetitive strain injuries included. Previously, we found that manual therapy treatments prevent inflammatory responses in nerve, muscle, and tendinous tissues; as well as somatosensory hypersensitivity, pathological neural discharge, and motor declines that develop with performance of repetitive overuse tasks (Bove et al., 2016, 2019; Barbe et al., 2021).

Unfortunately, avoiding repetitive work is not always possible, and preventive treatment is not routinely available. Thus, our goal in this study was to determine if a combination of commonly used forms of manual therapy applied to the forearm of unanesthetized rats could reverse the declines of repetitive strain injury, once established. Specifically, we examined the effectiveness of MT with rest to treat the sensorimotor declines and neuromuscular inflammation and fibrosis that had developed through the performance of a repetitive task.

## Materials and Methods

### Study Design

Three-arm randomized controlled trial.

### Animals

Experiments were approved by the Temple University Institutional Animal Care and Use Committee in compliance with NIH guidelines for the humane care and use of laboratory animals. Studies were conducted on 39 young adult (3months of age at onset), female, Sprague-Dawley rats (Charles Rivers, Wilmington, MA, United States). Female rats were used to allow comparison to our past studies on female rats using the same model and treatment (Bove et al., 2016, 2019; Barbe et al., 2019b).

Rats were numbered and randomly assigned to groups at the beginning of the study to ensure blinding. Rats were food restricted to weigh 5% less than age-matched rats with free access to food (these latter rats were used for weight comparison purposes only). This food restriction was necessary to motivate the rats to work for a food reward (Barbe et al., 2019a). Thereafter, all rats were carefully maintained at the 5% food restriction for the duration of the experiment. Food restricted control (FRC) rats received similar amounts of rat chow and food reward pellets as rats performing the task. All rats were weighed twice per week, provided regular rat chow daily in addition to food reward pellets [banana (F0024) and chocolate (F0165) grain-based dustless precision pellets, Bio-Serv, Flemington, NJ, United States], and allowed to gain weight over the course of the experiment as previously shown for this model (Massicotte et al., 2001; Jain et al., 2014; Smith et al., 2020; Barbe et al., 2021).

Twenty-nine rats were operatively shaped in custom-designed operant behavioral chambers to perform a high repetition and high force task (described below) for 14weeks before being randomly separated into three groups. One group was euthanized at this 14week TASK endpoint (TASK, *n*=10). The other two groups (*n*=19) of rats also ceased performing the task at this 14week time point. Nine were moved to a group that received manual therapy of their upper extremities, bilaterally, for 7weeks (MTR, manual therapy with rest), while 10 were assigned to rest alone for 7weeks (REST). Results were compared to FRC rats that did not perform the task and that did not receive manual therapy treatment (*n*=10), since we have previously reported that this treatment does not alter FRC rat behavioral or tissue outcomes (Barbe et al., 2021). FRC rats remained in their cages and were euthanized 26weeks after baseline, a time point equivalent to the 5weeks training+14weeks task+7weeks end point of treated TASK rats. The MTR, REST, and FRC groups continued to be food restricted during the final 7weeks, to allow comparison of results to the TASK group.

### Repetitive Task

The operant shaping lasted 5–6weeks during which the rats learned the reaching and lever-pulling task at high force loads (ramping upward from naïve, 10min/day, 5days/week), as previously described (Xin et al., 2017). Rats then performed a reaching and lever-pulling task for a food reward at a target grasp force of 1.39±0.01N, a mean grasp duration was 146±4.92ms, and a target reach rate of 4reaches/min, for 2h/day, in 30min intervals (with 1.5h break between session), for 3days/week (Barbe et al., 2020c). This repetitive task was performed for 14weeks by all rats, except the FRC rats. The experimental rats were allowed to and naturally do use either limb to pull on the lever bar, in an ambidextrous manner, across most training and task days.

### Manual Therapy

The treatment was a previously developed manual therapy protocol performed on unsedated rats (Bove et al., 2016) that included gentle forearm tissue mobilization, forearm skin rolling, a treatment intended to emulate “myofascial release” or “muscle stripping,” to the forearm flexor compartment, mobilization of the wrist joints, and a gentle traction (stretch and glide) to the entire upper extremity. We also added direct manipulation of the palm in this study, where the tip of the index finger was pressed into the palm of the rat, at and just distal to the transverse carpal ligament, with a rolling motion. The individual providing these treatments was a trained physical therapist (STP). Treatments were performed three times per week, on alternate days to the task performance, for 7weeks, on the MTR rats (*n*=9), and lasted approximately 5–7min per limb i.e., a total of 10–14min per rat.

### Examination of Indices of Potential Discomfort During the Manual Therapy Treatment

Because the manual therapy was provided to unsedated rats, there was opportunity to observe responsive behaviors. During and after each treatment session, the therapist recorded whether the rats withdrew or resisted lengthening of their forelimb in response to the treatment, and whether there were any behaviors indicative of increased stress (limb withdrawals, increased defecation, urination, attempts to escape, or biting). Mean results per week are reported. The therapist also recorded incidence of individual rat behaviors for 1h following treatment, looking for behaviors that could indicate pain or discomfort (paw guarding or licking, limping, excessive grooming, and movements in the cage).

### Sensorimotor Function Assay

Behavioral data from 39 rats in the study are reported. Since TASK rats use both limbs for reaching and pulling the lever bar, data are reported bilaterally for forepaw mechanical sensitivity and reflexive grip strength. For each test, the behavioral assays were performed by an examiner blinded to group assignment. For reflexive grip strength and cold temperature sensitivity, the testing devices’ software calculated the data, thus reducing any potential bias.

Forepaw mechanical sensitivity was assessed, bilaterally, using a 1g nylon monofilament (Semmes-Weinstein monofilaments, Stoelting) using previously described methods (Fisher et al., 2015). The number of responses out of 10 was recorded for each test session. The maximum score observed at baseline vs. at the end of the experiment (2–3days prior to tissue collection) was used to calculate the percent change for each individual rat and limb {[(endpoint value – baseline value) ÷ baseline value]×100}.

Reflexive grip strength was assayed using a rat grip strength meter (Columbus Instruments, Columbus, Ohio, United States), bilaterally (Barbe et al., 2013; Fisher et al., 2015). The maximum grip strength value observed at baseline or during the first 3weeks of training vs. at the end of the experiment (2–3days prior to tissue collection) was used to calculate the percent change in grip strength for each individual rat and limb, as previously utilized (Hilliard et al., 2021).

A forehead sticker removal test was used to determine functional agility and discomfort of each forelimb, using previously described methods (Kietrys et al., 2012). Briefly, forepaw and forelimb movements were scored as: 0=no attempt to remove the sticker, and 5=successful removal of the sticker. The maximum score observed at baseline vs. at the end of the experiment (2–3days prior to tissue collection) was used to calculate the percent change in forepaw/forelimb agility for each individual rat.

Cold temperature sensitivity was assessed using described methods (Fisher et al., 2015; Barbe et al., 2019b), once, a few days before euthanasia (to avoid confounds of learning the test) using a two-temperature choice apparatus (T2T system, Bioseb, Marseille, France). Briefly, rats were timed for how long they preferred to stand on a thermal plate set at room temp (22°C), as opposed to a second plate of decreasing temperature (22–12°C), in 2°C steps (3min per temperature step). The percent time spent on the variable test plate at each temperature step, vs. total time, was calculated from the system’s software. Only time spent on the12^o^C plate is reported.

### Median Nerve Electrophysiology

Because TASK rats use both limbs for reaching, a randomization scheme was used so that a mix of right and left limbs was collected for each assay choice (electrophysiology and histology vs. biochemical; Barbe et al., 2020a,b). Median nerve conduction velocity (NCV) was recorded immediately prior to euthanasia using a clinical electrophysiology device (UltraPro S100, NATUS Medical, Middleton, WI, United States), as previously described (Barbe et al., 2020a). Briefly, an orthodromic technique was used in which the median nerve was stimulated distally in the thenar eminence and sensory nerve action potentials recorded proximally at the elbow. NCV was derived from the peak of latency (ms) and is reported as m/s. Measurements were recorded at least five times per nerve. The average of these five recordings is reported.

### Collection of Median Nerves and Flexor Digitorum Muscles for Histological Assays

At 19weeks (TASK rats only) or 26weeks (remaining rats) after onset of the experiments and baseline testing, animals were deeply anesthetized with 5% isoflurane in oxygen. Depth of anesthesia was assessed and monitored by the pattern and rate of respiration, the absence of muscle tone, and the absence of toe and tail pinch, and eye blink reflexes. When the animals no longer showed any reflexive responses, an absence of muscle tone, and breathing had halted, the animals underwent a thoracotomy and cardiac puncture with exsanguination. This is a method of euthanasia that is in accordance with AVMA Guidelines for the Euthanasia of Animals.

Histological data were generated from nerves in the limb opposite of that used for ELISA from each of the 39 rats in the study. After collection of muscles from one limb each rat for biochemical assays, as described with ELISAs later, the rats underwent intracardial perfusion with first saline and then buffered 4% paraformaldehyde. After fixation, the median nerve, flexor digitorum muscle, and surrounding tissues still intact, were separated from forelimb bones with a scalpel. These pieces were immersion fixed for 48h in buffered 4% paraformaldehyde before being cryopreseserved in 10% and then in 30% sucrose in phosphate buffer (48h per sucrose solution). Tissues were embedded on dry ice in Optimum Cutting Temperature compound (23,730,571, FisherScientific, Houston, TX, United States) and cryosectioned into 14μm thick longitudinal serial slices (every section was collected on approximately 50 slides total). Sections were placed onto charged slides, with two sections per slide (22,037,200, FisherScientific, Pittsburgh, PA, United States), dried overnight at room temperature, before storage in foil-wrapped slide boxes at −80°C until use.

### Histological and Immunohistochemical Staining of Median Nerves

Subsets of slides were stained for: (1) activated macrophages, (2) degraded myelin basic protein (dmbp), or (3) collagen using a Masson’s Trichrome stain. For this, every 15th slide was pulled from the −80°C freezer, with three slides stained per rat, in batched sets, for a specific antibody or stain. Adjacent slides were used for the other antibody or stain. One subset of slides was immunostained with a specific antibody against CD68/ED1 (ab31630, Abcam; a mouse anti-rat monoclonal antibody that recognizes a single chain glycoprotein of 110kDa expressed predominantly on the lysosomal membrane of myeloid cells; recognizes an ED1 protein that is the rat homologue of human CD38; Al-Shatti et al., 2005; Roszer, 2015; Pinto et al., 2018) at 1:300 dilution in phosphate buffered saline (PBS). A second subset of slides was immunostained using a rabbit anti-rat polyclonal antibody against dmbp (recognizes myelin basic protein in demyelinated nerve tissues; AB5864, Millipore Sigma, Burlington, Massachusetts, United States) at 1:500 dilution in PBS. Additional sections were double-labeled with the anti-CD68 antibody and a rabbit anti-rat polyclonal antibody against tumor necrosis factor alpha (TNFα; AB1837P, Millipore Sigma) at 1:250 dilution in PBS for overnight at room temperature (after similar pepsin digestion and goat serum pre-treatments). The tissue sections, on slides, to be immunostained with antibodies first underwent 0.5% pepsin digestion in 0.01N HCl for 15min at room temperature, and then a 30min incubation in 10% goat serum to reduce non-specific binding. Sections were then incubated with one of the primary antibodies for overnight at room temperature. The sections were then washed in PBS (three times, 5min each), followed by incubation with the appropriate secondary antibody conjugated to green or red fluorescent tags (AF488 or Cy3; Jackson ImmunoResearch, West Grove, PA, United States) at a dilution of 1:100 each for 2h at room temperature. 4',6-diamidino-2-phenylindole (DAPI) was used as a nuclear counterstain following the immunostaining. The immunofluorescent stained slides were coverslipped with 80% glycerol in PBS before visualization.

The CD68 antibody used was previously validated using immunohistochemical methods (no primary antibody controls in which serum was substituted for the primary antibody, followed by secondary antibodies; no labeling was observed). The antibody against degraded myelin basic protein used was similarly validated (no primary antibody controls in which serum was substituted for the primary antibody, followed by secondary antibodies; no labeling was observed; Barbe et al., 2020a). Both were further verified by staining FRC rat nerve sections with the antibodies and findings of few to no CD68+ cells or degraded myelin basic protein staining in the nerves of these rats.

The third subset of slides underwent Masson’s Trichrome staining, which stains collagen blue. The slides were dehydrated through increasing concentration of ethanol, cleared with xylene, and then coverslipped with DPX mountant (06522, Sigma-Aldrich).

### Quantification of Staining/Immunostaining of Median Nerves

The individual performing the image analyses were naïve to group assignment (rats had been previously numbered to blind group assignment). The imaging and assessments were carried out by one person experienced in these methods (MFB). Imaging was performed using an epifluorescent microscope (E1000; Nikon, Melville, NY, United States) with a motorized stage that was interfaced with a digital camera (Retiga 4000R QImaging Firewire Camera, Surry, BC, Canada) and a bioquantification software system (Life Science 2020, Bioquant, Nashville, TN, United States).

To quantify the number of CD68+ immunoreactive cells in the median nerve, prior to the counts of each image, the gain and exposure of the camera were set and then maintained consistently throughout. Three different sections per rat forelimb that contained the median nerve at the level of the wrist were chosen for analysis (typically 196μm distant from each other). Three different microscope fields were assayed per nerve using a 20X objective. Counts were made only within the boundaries of the sectioned median nerve in a 0.07mm^2^ or smaller field (as depicted in Al-Shatti et al., 2005; Bove et al., 2019) using the Irregular Region of Interest tool of the Bioquant software. Only regions inside the epineurium were assayed. Then, the number of CD68+ cells (green) containing a DAPI stained nucleus was counted, per region of interest and per field, using the select object count option of the Bioquant software. The numbers of immunostained CD68+ cells with DAPI+ nuclei were normalized to the mm^2^ of the area of the median nerve in the imaged field.

Degraded myelin basic protein was assayed in the same approximate region using the same microscope and imaging system using a 20X objective and a thresholded pixel count (percentage of stained pixels in the region of interest; Al-Shatti et al., 2005; Barbe et al., 2020a). In detail, three different microscope fields were measured per median nerve at the point that it crossed the carpal tunnel, in three sections per rat, as described for CD68 staining. Measurements were made only within the boundaries of the sectioned median nerve in a 0.07mm^2^ or smaller field (as shown in Clark et al., 2003; Al-Shatti et al., 2005) using the Irregular Region of Interest tool. The Videocount Area Array option of the Bioquant software was utilized for the thresholded measurements. Videocount area is defined as the number of pixels in a field that meet a user-defined color threshold of staining multiplied by the area of a pixel at the selected magnification. Color threshold values were first selected based on mean levels of red dmbp immunostaining in experimental animal nerve sections (there was little to no dmbp staining in the FRC rat nerves; thus defining the bottom limit of the threshold). The threshold values were then stored in the computer program for consistent automeasurement of the immunostained slides. The number of pixels with degraded myelin basic protein immunostaining was then quantified in all nerve images using this consistent range of threshold values (and consistent camera and image capture settings). The percent area fractions of dmbp immunoreaction product were then calculated by dividing the videocount area containing pixels within the defined thresholds, by the videocount area of the total number of pixels in the region of interest, and multiplying by 100.

Extraneural collagen deposition around nerves was quantified in Masson’s Trichrome stained slides, using the same imaging system and previously described methods (Bove et al., 2016; Barbe et al., 2019a). First, the microscope’s light intensity (bright field), camera gain and exposure were standardized and remained constant. The range for the blue color stain was determined; this threshold was saved in a subprogram, and then applied to all images for consistent auto-measurement of the blue stain. The number of thresholded blue pixels in each field were counted using a 20X objective and the imaging system to visualize the nerves, and analyzed as a percentage of the total number of pixels in that field. This was performed in three fields per nerve, and three sections per animal.

### Collection of Forearm Flexor Digitorum Muscles for ELISA

From one limb per rat, mid-to proximal regions of the flexor digitorum muscles were separated from bones and then tendons, rinsed in sterile saline, dissected into smaller samples of about 50–100mg each, and flash frozen in liquid nitrogen before storage at −80°C. As previously described (Iannarone et al., 2019; Barbe et al., 2019a), frozen muscle samples were homogenized in sterile, ice-cold, phosphate-buffered saline (PBS) containing fresh proteinase inhibitors (cOmplete™ EDTA free Protease Inhibitor tablets, 5,056,489,001, Sigma-Aldrich, Inc., St. Louis, MO, United States). Homogenated lysates were centrifuged at 12,000rpm for 15min at 4°C. Lysate supernatants were aliquoted and stored at −80°C until assayed *via* ELISA, in duplicate. Muscle lysate supernatants from one limb of eight rats/group were assayed for TNFα and interleukin 10 (IL-10) using manufacturers’ protocols (TNFα kit was EA100366, Origine Technologies, Inc., Rockville, MD, United States; IL-10 kit was BMS629, Affymetrix/ebioscience, Vienna, Austria). Additionally, muscle lysate supernatants from one limb of 9–10 rats/group were assayed for collagen type I (LS-F5638, LifeSpan BioSciences, Inc., Seattle, WA, United States), using manufacturers’ protocols. Data for each ELISA outcome (pg of protein) were normalized to μg total protein, determined using a bicinchoninic acid protein assay kit (23,227, BCA Protein assay, Pierce, ThermoFisher Scientific, Rockford, IL, United States).

### Statistical Analyses

GraphPad Prism version 8.2 (GraphPad, San Diego, CA, United States) was used for the statistical analyses, and figure assembly was performed using Adobe Photoshop CC 2017. The sample size for this study was derived from our previous studies (Bove et al., 2016), assuming a power of 80% and level of significance of 0.05, and a SD of 28 for grip strength. Data are represented graphically as mean±95% CI. Exact *p* values are reported for all data with a minimum of 0.05 being considered statistically significant. The *p* values reported for the *post hoc* findings are adjusted for the number of multiple comparisons performed. The percent withdrawal responses to manual therapy treatment were assayed using a Freidman test, followed by Dunn’s multiple comparisons *post hoc* test. Both Shapiro-Wilk and Kolmogorov-Smirnov tests of normality were performed, and residuals were inspected. Data for cold temperature sensitivity were not normally distributed, so Kruskal-Wallis nonparametric tests were used to compare data between groups, and *post hoc* testing using the two-stage step-up method of Benjamini et al. (2006). A repeated measures mixed effects model (REML, Restricted Maximum Likelihood) with Geisser-Greenhouse corrections was used to compare percent change from baseline for forepaw mechanical sensitivity, reflexive grip strength, and forepaw/forelimb agility data, using two factors (behavior and treatment group), followed by the two-stage step-up method of *post hoc* test of Benjamini et al. (2006). The NCV, nerve histological data and muscle ELISA data were normally distributed, so one-way ANOVAs were used for each, followed by the two-stage step-up method of *post hoc* testing of Benjamini et al. (2006). Spearman’s rank correlation tests were used to determine correlations between various outcomes using all data from all rats in the study in an *XY* setup in which there were multiple *Y*’s to each *X* (for example, if nerve CD68+ cells was *X*, then the *Y*’s were: % change in response to a 1g monofilament, grip strength, or forepaw/forelimb agility, NCV, and temperature sensitivity to 12°C). Values between 0.3 and 0.59 (−0.3 and−0.59) were considered as a moderately positive (or negative) relationships, and values between 0.6 and 0.79 (−0.6 and−0.79) as a strongly positive (or negative) relationships, and +/− 0.8–1.0 as very strong correlations (Swinscow, 1997; Lamorte, 2019). Only moderate to very strong correlations were considered and reported in the text of the results, although these are rather arbitrary limits, and the context of the results should be considered. All correlation findings are reported in a Table.

## Results

### Tolerance of the Manual Therapy Treatment

**Figure 1:**
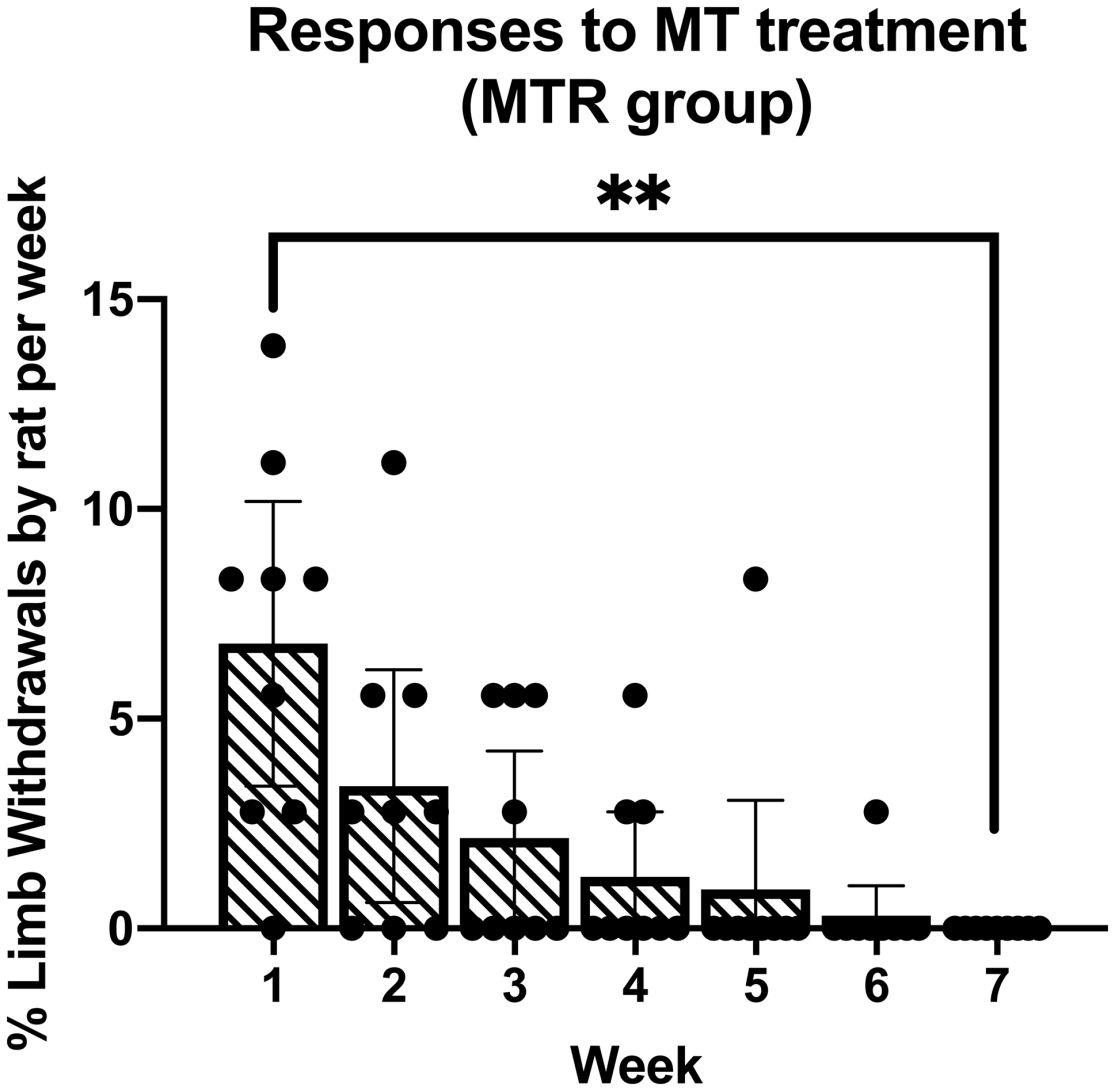
Responses of the manual therapy with rest (MTR) rats to the manual therapy across 7weeks of treatment. The mean percent limb withdrawal by rat per week of treatment is presented. ^**^p<0.01, compared to week 1 of treatment. Mean±95% CI shown.

As in previous studies (Bove et al., 2016, 2019; Barbe et al., 2021), the manual therapy treatment used was well tolerated by the animals. An initial increase in percent limb withdrawal during treatment of the MTR animals in week 1 resolved with continued treatments across the weeks (Figure 1). By week 7, the percent limb withdrawal during treatment was significantly lower than in week 1 (*p*=0.001). There were no aggressive behaviors, such as attempts to escape or biting throughout the experiment, and no post-treatment behaviors indicative of discomfort or stress.

### Effects on Sensory and Motor Related Behavior

Three sensorimotor behaviors were assayed at baseline and in the final week of task or treatment, including forepaw skin sensitivity to probing with a 1g monofilament, reflexive grip strength, and the forehead sticker removal test (Kietrys et al., 2012; Hadrevi et al., 2019). The percent change from baseline was calculated, as previously utilized (Hilliard et al., 2021), and the three data sets were analyzed together using a repeated measures mixed model analysis. This revealed significant effects for behavior (*p*<0.0001, *F*_2,153_=15.13), treatment group (*p*=0.04, *F*_3,177_=2.82), and their interaction (*p*<0.0001, *F*_6,177_=10.52).

**Figure 2:**
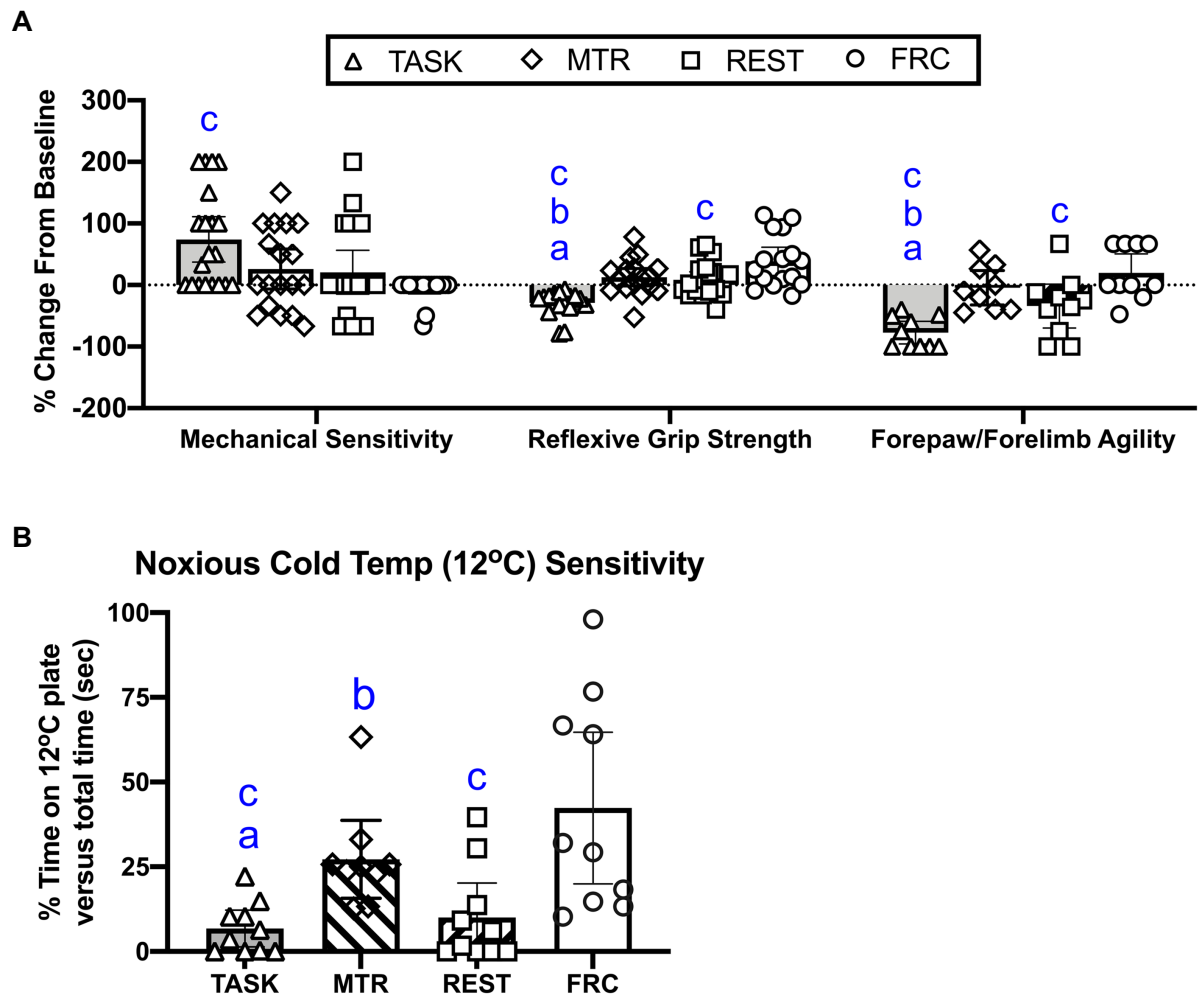
Sensorimotor behavioral changes. **(A)** From left to right, percent change from baseline in forepaw mechanical sensitivity to probing with a 1g monofilament, reflexive grip strength, and forepaw/forelimb agility. **(B)** Sensitivity to 12°C. Symbols: *a*, *b*, and *c*: *p*<0.05 each, compared to MTR, rest alone (REST), and Food restricted control (FRC) groups, respectively. Mean±95% CI shown.

As shown in Figure 2A, left side, *post hoc* analysis showed that TASK rats showed a higher percent increase from baseline in forepaw mechanical in task week 14, compared to the FRC group (*p*=0.0002), indicative of enhanced forepaw skin sensitivity in the TASK rats. There were no differences between the MTR, REST, and FRC groups. Regarding reflexive grip strength (Figure 2A, middle), *post hoc* analysis showed that TASK rats showed a greater percent decrease from baseline, compared to the other groups (*p*<0.0001 each). The REST group also showed a greater percent decrease in grip strength, compared to the FRC group (*p*=0.02). There were no differences between the MTR and REST, or MTR and FRC groups. Regarding the rats’ forepaw/forelimb agility (Figure 2A, right side), *post hoc* analyses showed that TASK rats had a significant decrease in agility from their baseline, compared to MTR (*p*=0.002), REST (*p*=0.02), and FRC (*p*<0.0001) groups. The REST group also showed a significant decrease in forepaw/forelimb agility, compared to the FRC group (*p*=0.02). There were no differences between the MTR and REST, or MTR and FRC groups. These results are summarized in Table 1.

**Table 1:** Summary of data by type and with brief interpretation.

Assay	Different from Task?			Different from REST?		Interpretation
	MTR	REST	FRC	MTR	FRC	
**Behavior**
Mechanical sensitivity (Figure 2A)	No	No	Less	No	No	No effect
Reflexive grip strength (Figure 2A)	Stronger	Stronger	Stronger	No	Slightly Stronger	Both treatments improved grip; Rest provided less improvement
Forepaw/Forelimb agility (Figure 2A)	More	More	More	No	More	MTR attenuated agility deficit; Rest provided less improvement
Noxious cold sensitivity (i.e., aversion; Figure 2B)	Less	No	Less	**Less**	Less	**MTR ameliorated cold sensitivity more than REST**
Nerve conduction velocity (Figure 3)	Faster	No	Faster	No	Faster	MTR attenuated NCV declines; Rest provided less improvement
**Median nerve**
CD68/ED1+ macrophages numbers (Figures 4A, 5)	Fewer	Fewer	Fewer	No	No	Both treatments rescued macrophage infiltration
Degraded myelin basic protein (Figures 4B, 5)	Less	Less	Less	No	No	Both treatments rescued myelin degeneration
Collagen/Fibrosis (Figures 4C, 6)	Less	No	Less	Trend to less (*p*=0.06)	Less	MT attenuated nerve collagen deposition, while REST did not
**Flexor Digitorum Muscle**
TNFα (Figure 8A)	Less	Less	Less	No	No	Both treatments reduced muscle TNFα
IL-10 (Figure 8B)	More	More	No	**More**	More	**MTR increased IL-10 more than REST**
Collagen type I (Figure 8C)	Less	Less	Less	No	No	Both treatments reduced muscle Collagen I production

Significant differences between MTR and REST groups are bolded. CD68/ED1, an activated macrophage marker; IL-10, interleukin 10; macs, macrophages, MT, manual therapy; NCV, nerve conduction velocity; and TNFα, tumor necrosis factor alpha.

Cold temperature sensitivity was assessed by examining the percent time that rats spent on a noxious cold temperature plate (12°C) vs. total time. A significant difference between groups was observed (*p*=0.005, Kruskal-Wallis 17.85). *Post hoc* comparisons showed that the TASK rats were more sensitive to 12°C, compared to the MTR group (*p*=0.005), as were the REST rats (*p*=0.01, compared to the MTR group; Figure 2B). The TASK and REST groups were also more sensitive to 12°C than the FRC group (*p*=0.0007 and *p*=0.002, respectively). There were no differences between the MTR and FRC groups. See Table 1.

### Effects on Median NCVs

**Figure 3:**
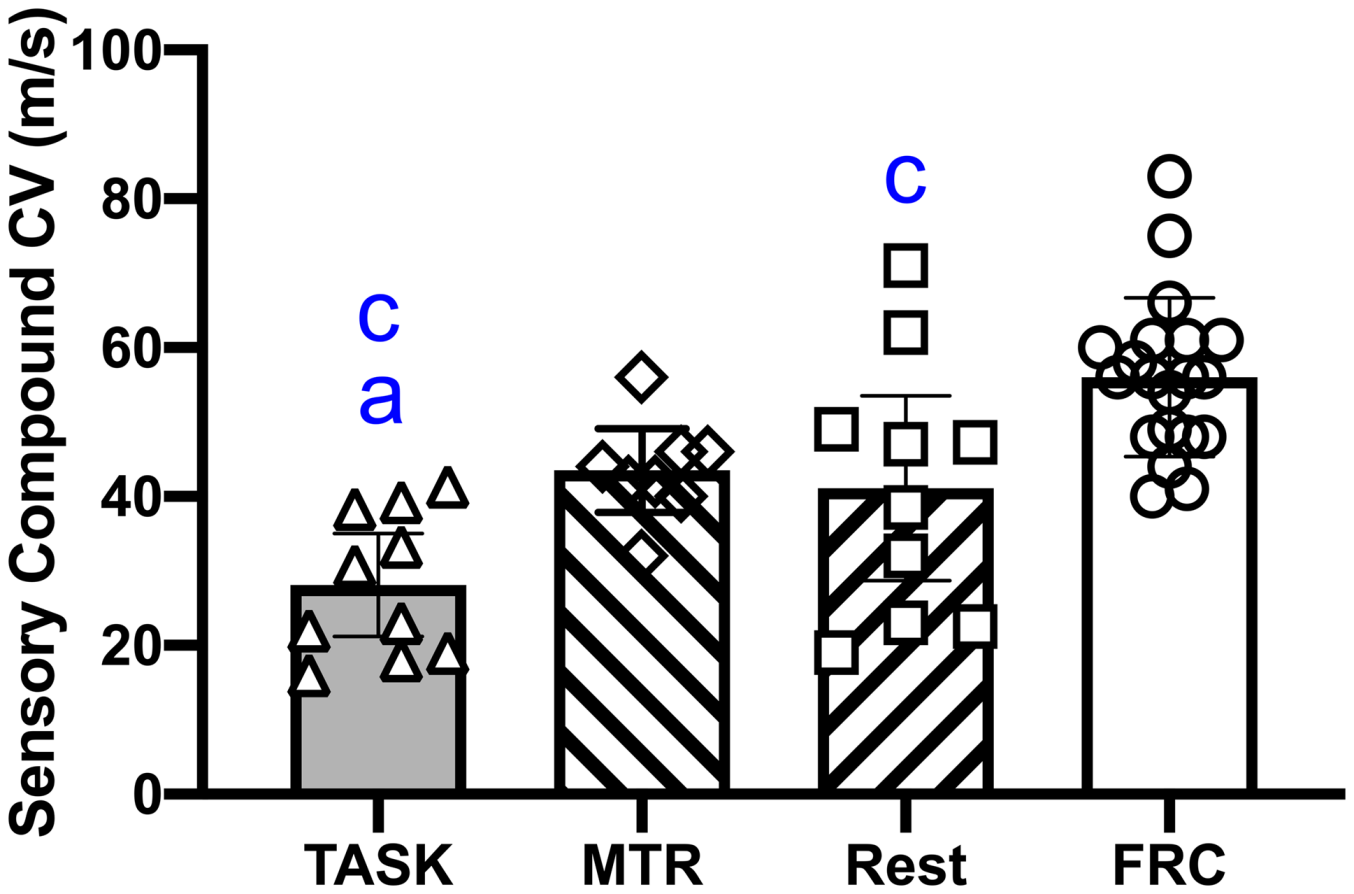
Median nerve conduction velocity (NCV). Symbols: *a* and *c*: *p*<0.05 each, compared to the MTR and FRC groups, respectively. Mean±95% CI shown.

Median nerve function was examined *in vivo* using an orthodromic method, as previously utilized (Barbe et al., 2020a). A one-way ANOVA showed a significant difference between groups (*p*<0.0001; *F*_3,44_=13.29). *Post hoc* comparisons showed that the median nerves of TASK rats had slower NCVs than the MTR and FRC rats (*p*=0.04 and *p*<0.0001, respectively, Figure 3). The REST rats also had slower NCVs than FRC rats (*p*=0.01). There were no differences between the MTR and REST, or MTR and FRC groups. See Table 1.

### Effects on Median Nerve Inflammatory Macrophages, Degraded Basic Myelin Protein, and Collagen Deposition

**Figure 4:**
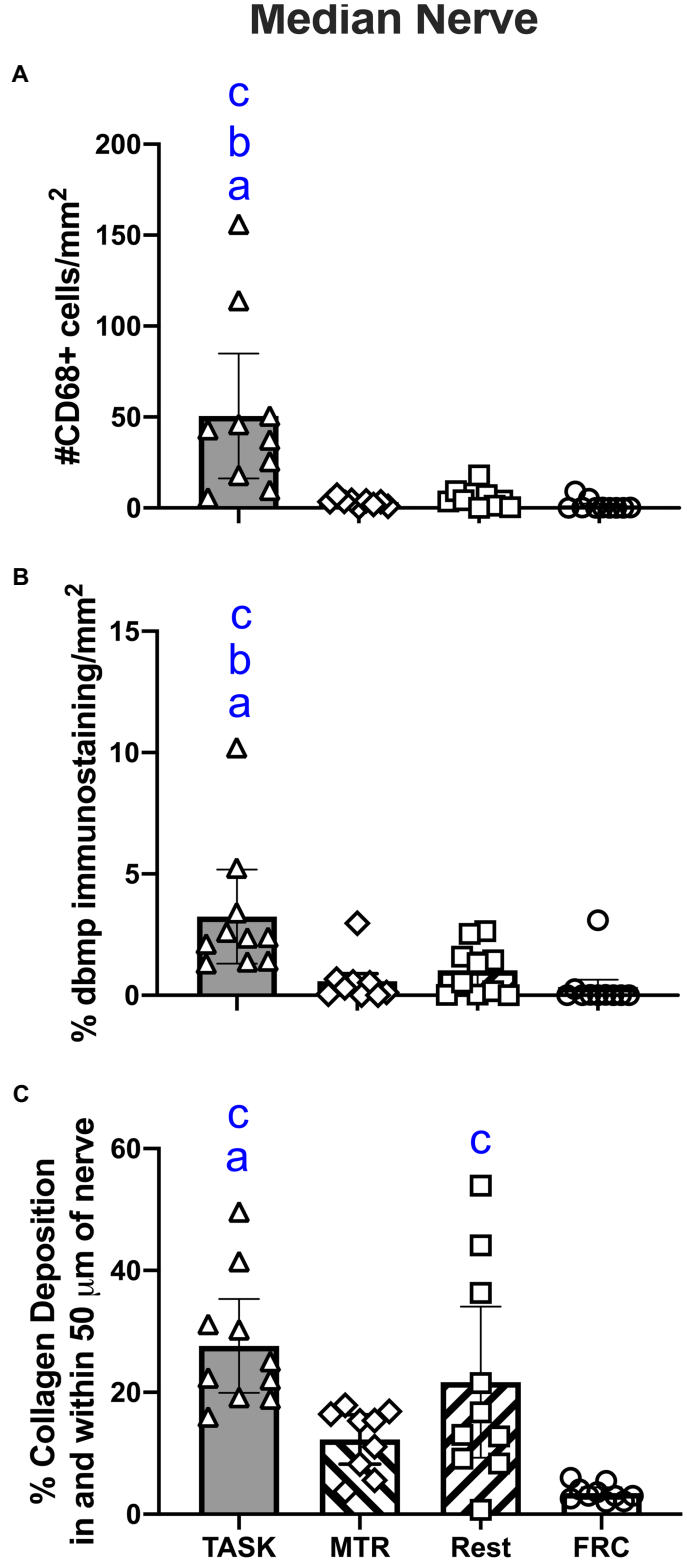
Median nerve histomorphometry, at the level of the wrist. **(A)** Number of CD68+ macrophages. **(B)** Percent degraded myelin basic protein (dmbp) immunostaining in nerve. **(C)** Percent collagen deposition/fibrosis in and around the nerve in Masson’s Trichrome stained sections. Symbols: *a*, *b*, and *c*: *p*<0.05 each, compared to MTR, REST, and FRC groups, respectively. Mean±95% CI shown.

Median nerves were assayed for inflammation by counting the number of CD68/ED1 immunopositive cells in the nerve at the level of the wrist. A one-way ANOVA showed a difference between groups (*p*=0.0001, *F*_3,35_=9.17). *Post hoc* analyses showed increased numbers of CD68+ macrophages in the TASK group, compared to MTR, REST, and FRC groups (*p*=0.0002, *p*=0.0002, and *p*<0.0001, respectively; Figure 4A). There were no differences between the MTR, REST, or FRC groups. Adjacent sections were assayed for nerve degradation by quantifying immunoexpression of degraded myelin basic protein in the nerve at the level of the wrist. A one-way ANOVA showed a difference between groups (*p*=0.001, *F*_3,35_=6.71). *Post hoc* analyses showed more degraded myelin basic protein in the TASK group, compared to MTR, REST, and FRC groups (*p*=0.001, *p*=0.004, and *p*=0.003, respectively; Figure 4B). There were no differences between the MTR, REST, or FRC groups. Another set of adjacent sections was assayed for intraneural and extraneural fibrosis after Masson’s Trichrome staining, and quantification of the percent collagen staining. A one-way ANOVA showed a significant difference between groups (*p*<0.0001, *F*_3,35_=9.81). *Post hoc* analyses showed more nerve fibrosis in the TASK group, compared to the MTR and FRC groups (*p*=0.004 and *p*<0.0001, respectively; Figure 4C). REST rats had more neural fibrosis than FRC rats (*p*=0.005), and there was a trend toward a difference between the REST and MTR groups (*p*=0.06). Collagen deposition in and around MTR nerves was similar to that seen in FRC nerves.

These results are summarized in Table 1.

**Figure 5:**
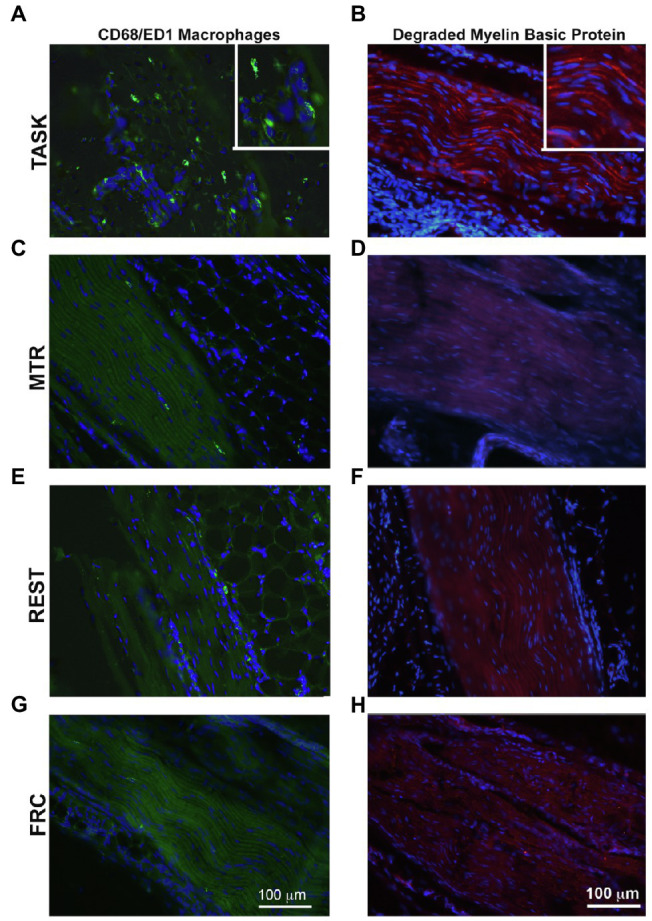
CD68-immunopositive macrophages (green) and degraded myelin basic protein (red) in longitudinal sections of the median nerve. 4',6-diamidino-2-phenylindole (DAPI) was used as a counterstain (blue). **(A,B)** TASK rats. **(C,D)** MTR rats. **(E,F)** Rest rats. **(G,H)** FRC rats. Left panels: Representative images of CD68+ cells in median nerves at wrist level. Right panels: Representative images of degraded myelin basic protein in median nerves at wrist level. Scale bars in panels **A** and **B**=100μm and applies to the other panels.

**Figure 6:**
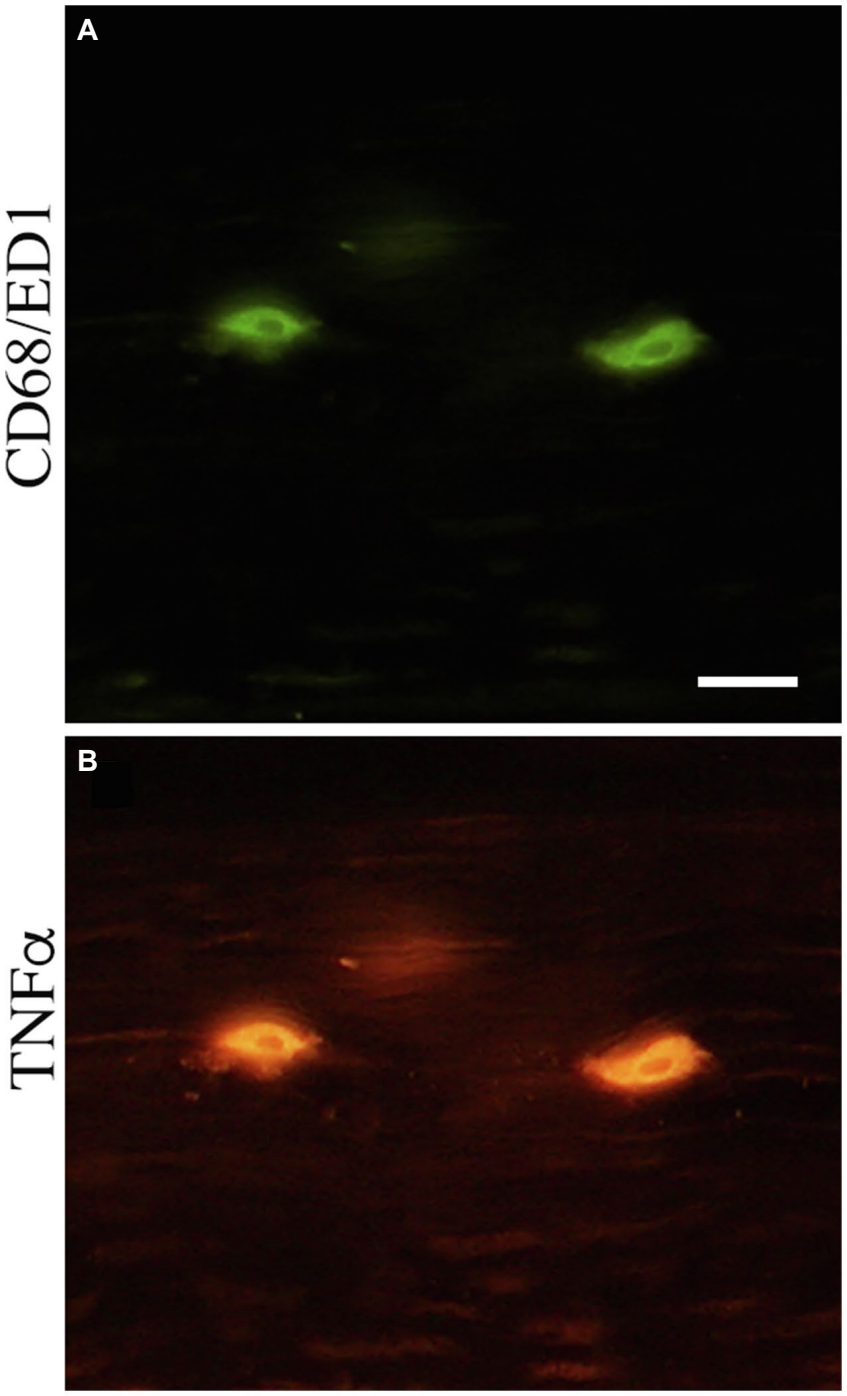
CD68-immunopositive cells double-labeled with tumor necrosis factor alpha (TNFα) in the median nerve at wrist level. **(A)** CD68-immunopositive cells (green). **(B)** TNFα immunopositive cells (red). Scale bar=50μm.

Representative images of these median nerve changes are shown in Figure 5. CD68+ macrophages and degraded myelin basic protein were prevalent within median nerves of TASK rats (Figures 5A,B). The insets of Figures 5A,B show higher power images of the CD68+ macrophages and degraded myelin basic protein in TASK rat nerves. These inflammatory and degradative nerve changes were resolved in the median nerves of MTR and REST rats (Figures 5C–F), which resembled FRC nerves (Figures 5G,H). Figure 6 shows that these CD68+ macrophages also expressed TNFα.

**Figure 7:**
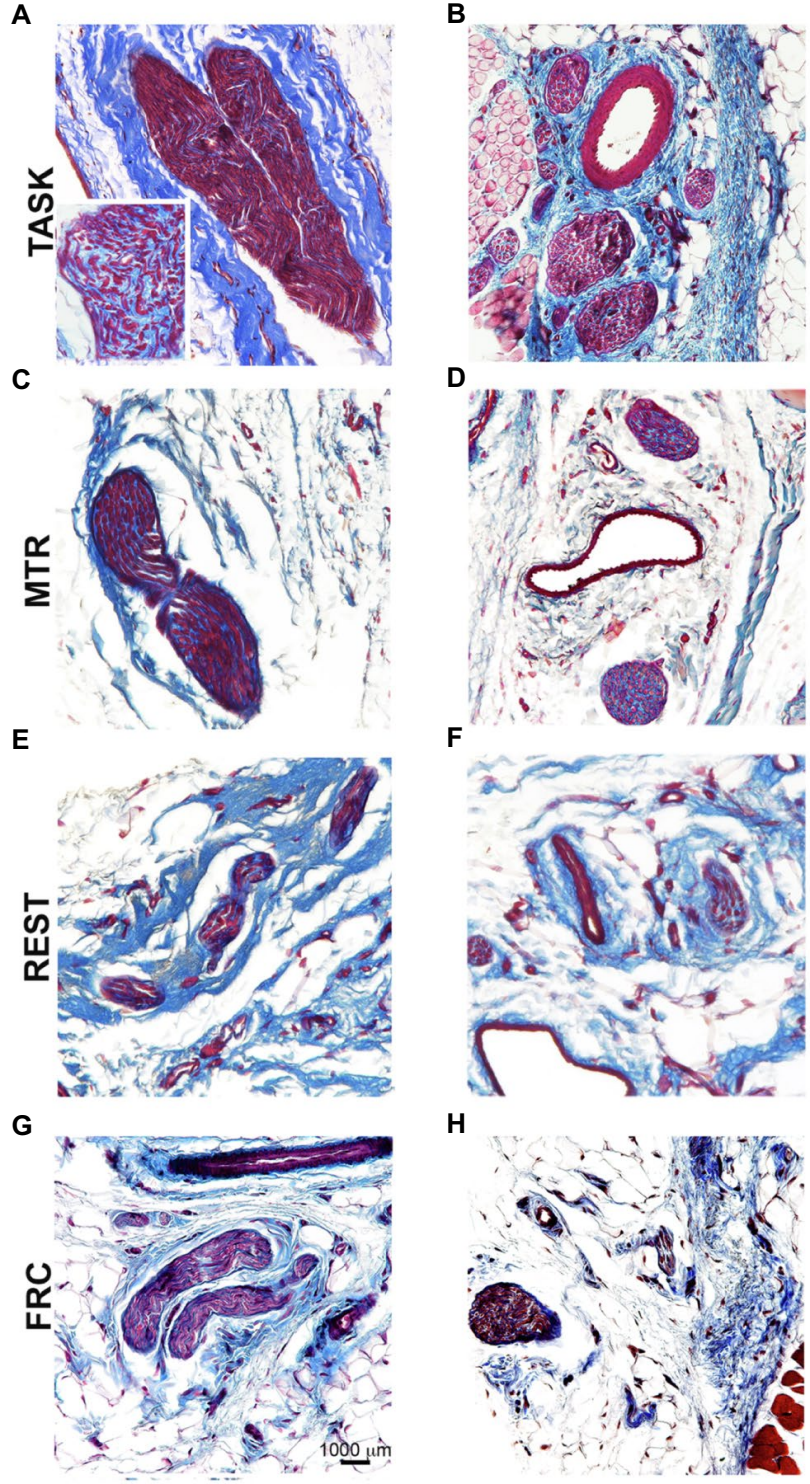
Masson’s Trichrome staining for collagen in and around median nerves at wrist level. **Left panels** **(A,C,E,G)**: Representative images of longitudinal sections of the median nerve. **Right panels** **(B,D,F,H)**: Representative images of cross-sections of the median nerve. Scale bar in panel **G**=1,000μm and applies to the other panels.

Representative images of median nerve collagen deposition after staining with Masson’s Trichrome are shown in Figure 7. Intraneural and extraneural fibrosis, detected as enhanced collagen (blue staining), was prevalent within and around median nerves at the level of the wrist in TASK rats (Figures 7A,B). These fibrotic nerve changes were resolved in the median nerves of MTR rats (Figures 7C,D), which resembled FRC rat nerves (Figures 7G,H). Enhanced collagen staining was still present around REST rat nerves (Figures 7E,F).

### Correlations Between Median Nerve Immunohistological/Histological Responses and Function

Spearman’s rank correlation tests were used to determine correlations between nerve immunohistological/histological responses and functional findings. Numbers of CD68+ macrophages in the median nerve at wrist level correlated: (1) moderately and positively with percent change in forepaw sensitivity (*r*=0.37, *p*=0.01); (2) strongly and negatively with percent change in forepaw/forelimb agility (*r*=−0.67, *p*=0.004) and NCV (*r*=−0.66, *p*=0.0008); and (3) moderately and negatively with percent change in grip strength (*r*=−0.53, *p*=0.0007) and 12°C cold sensitivity (*r*=−0.45, *p*=0.008). Immunoexpression of degraded myelin basic protein in the median nerve correlated moderately and negatively with percent change in percent change in grip strength (*r*=−0.48, *p*=0.006), forepaw/forelimb agility (*r*=−0.64, *p*=0.01), and 12°C cold sensitivity (*r*=−0.47, *p*=0.02). Collagen staining in and around the median nerve correlated: (1) strongly and negatively with percent change in forepaw/forelimb agility (*r*=−0.66, *p*=0.005); and (2) moderately and negatively with 12°C cold sensitivity (*r*=−0.43, *p*=0.04) and NCV (*r*=−0.48, *p*=0.03). Combined, these results suggest that median nerve inflammation, degradation, and fibrosis are strong contributors to the sensorimotor and nerve function results observed. All correlation findings are listed in Table 2.

**Table 2:** Spearman’s rank correlations between median nerve immunohistochemical and histological responses and functional outcomes.

Variables	% Change in forepaw sensitivity	% Change in grip strength	% Change in forepaw/forelimb agility	Noxious cold sensitivity (aversion)	NCV
**Median nerve**					
CD68/ED1+ macrophages	**0.37 (*p* =0.01)**	−**0.53 (*p* =0.0007)**	**−0.67 (*p* =0.004)**	−**0.45 (*p* =0.008)**	−**0.66 (*p* =0.0008)**
Degraded myelin basic protein	0.007 (*p* =0.49)	−**0.48 (*p* =0.006)**	−**0.64 (*p* =0.01)**	**−0.47 (*p* =0.02)**	0.12 (*p* =0.31)
Collagen/Fibrosis	0.26 (*p* =0.07)	−0.29 (*p* =0.07)	−**0.66 (*p* =0.005)**	**−0.43 (*p* =0.04)**	−**0.48 (*p* =0.03)**
**Flexor digitorum muscle**					
TNFα	0.29 (*p* =0.22)	**−0.42 (*p* =0.02)**	−0.22 (*p* =0.22)	0.02 (*p* =0.47)	**−0.50 (*p* =0.03)**
IL-10	−0.02 (*p* =0.47)	0.26 (*p* =0.12)	**0.57 (*p* =0.03)**	−0.12 (*p* =0.31)	−0.06 (*p* =0.40)
Collagen type I	0.21 (*p* =0.23)	0.05 (*p* =0.45)	−0.20 (*p* =−0.32)	0.10 (*p* =0.32)	−0.12 (*p* =0.42)

Significant findings are bolded. 0.3–0.59: moderate correlation; 0.6–0.79: strong correlation; 0.8–1.0: very strong correlation; “+” sign indicates positive correlation; “−” sign indicates negative correlation. Other abbreviations as defined in Table 1.

### Effects on Muscle Levels of TNFα, IL-10, and Collagen Type I

**Figure 8:**
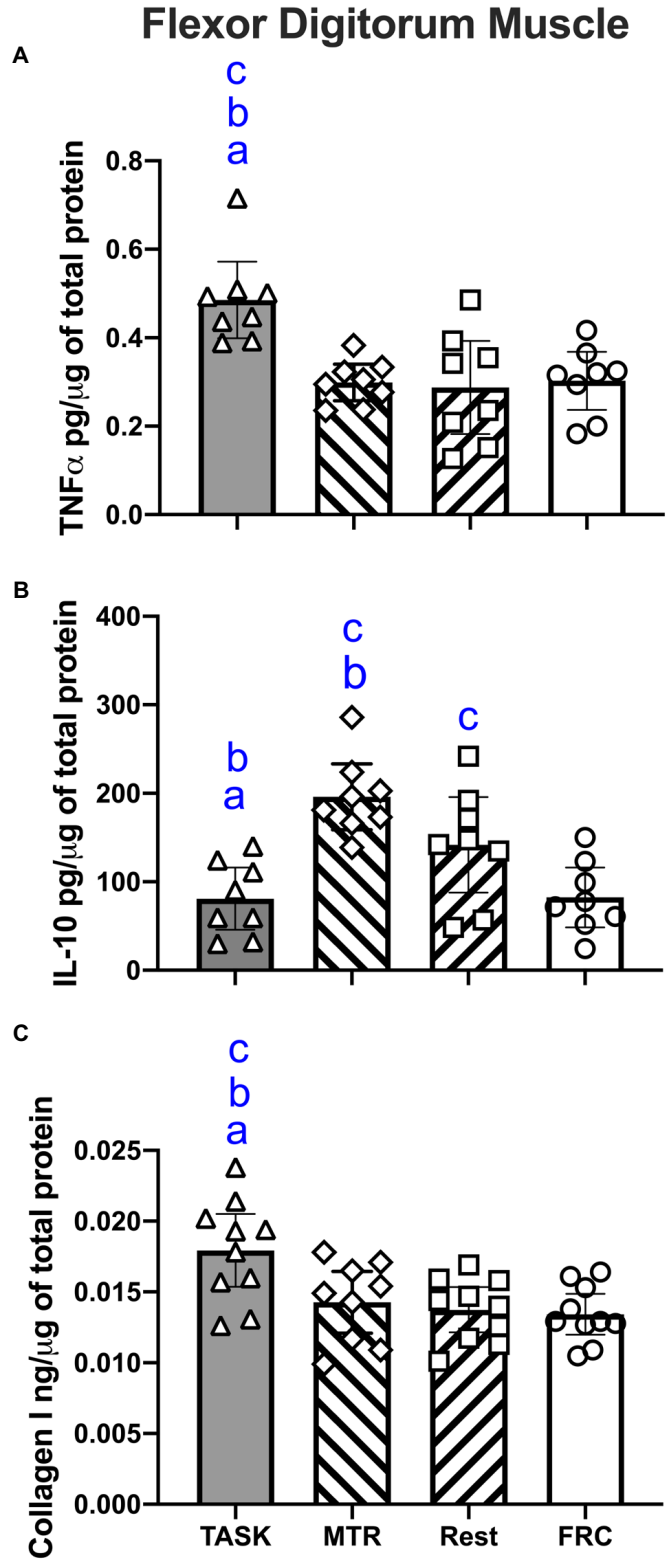
ELISA results for flexor digitorum muscle lysates. **(A)** TNFα levels. **(B)** IL-10 levels. **(C)** Collagen type I levels. Symbols: *a*, *b*, and *c*: *p*<0.05 each, compared to MTR, REST, and FRC groups, respectively. Mean±95% CI shown.

A one-way ANOVA showed that TNFα levels, a key pro-inflammatory cytokine, differed across groups (*p*=0.0005; *F*_3,28_=8.18). *Post hoc* analyses showed higher TNFα levels in TASK rat muscles, compared to MTR, REST, and FRC groups (*p*=0.0004, *p*=0.0002, and *p*=0.0005, respectively; Figure 8A). There were no differences between the other groups.

A one-way ANOVA showed a significant difference in IL-10 muscle levels, a key anti-inflammatory cytokine, across the groups (*p*=0.0001; *F*_3,28_=10.15). In contrast to TNFα findings, *post hoc* analyses showed the highest levels of IL-10 levels were in MTR muscles, compared to TASK, REST, and FRC muscles (*p*<0.0001, *p*=0.03, and *p*<0.0001, respectively; Figure 8B). Also, REST rat muscles had higher levels than TASK and FRC muscles (*p*=0.02 each).

A one-way ANOVA showed a significant difference in collagen type I muscle levels, a key fibrosis related protein, between groups (*p*=0.002; *F*_3,35_=5.84). *Post hoc* analyses showed higher collagen type I levels in the TASK muscles, compared to MTR, REST, and FRC muscles (*p*=0.006, *p*=0.002, and *p*=0.008, respectively; Figure 8C). There were no differences between the other groups.

These results are summarized in Table 1.

### Correlations Between Muscle Inflammation and Fibrosis Responses and Function

Spearman’s rank correlation tests showed moderate and negative correlations between muscle levels of TNFα and the percent change in reflexive grip strength (*r*=−0.42, *p*=0.02) and NCV (*r*=−0.50, *p*=0.03). Spearman’s rank correlation tests showed moderate and positive correlations between muscle levels of IL-10 and the percent change in forepaw/forelimb agility (*r*=0.57, *p*=0.03). There were no significant correlations between muscle collagen levels and any functional outcome. All correlation findings are listed in Table 2.

## Discussion

TASK rats that performed the repetitive work for 14weeks showed progressive sensory and motor declines, and multiple neuromuscular pathologies that resemble many of those seen in humans with upper extremity repetitive strain injuries or median mononeuropathy (Borg and Lindblom, 1988; Carp et al., 2007; Fernández-De-Las-Peñas et al., 2016; Wolny et al., 2017; Wendt et al., 2018; Singh and Srivastava, 2020; Zimmerman et al., 2020). Specifically, TASK rats showed enhanced mechanical sensitivity of the forepaw, greater sensitivity of a noxious cold temperature, declines in reflexive grip strength and forelimb/forepaw agility, and reduced median NCV. On a tissue level, prolonged task performance induced median nerve inflammation, myelin degradation, extraneural fibrosis, muscle inflammation, and increased muscle collagen production. We sought to determine if manual therapy concurrent with rest would reverse these deleterious responses. Using a 7-week manual therapy treatment that we have shown prevents these changes from developing (Bove et al., 2016, 2019; Barbe et al., 2021), we observed more improvement than rest alone in metrics indicative of neuropathy (forepaw/forearm agility, cold sensitivity, slow median NCV, and neural fibrosis), compared to TASK rats, and an amelioration of cold sensitivity that remained in REST rats (see Table 1). Muscle levels of IL-10, a potent anti-inflammatory cytokine, increased more with manual therapy than with rest alone, a change that benefited grip strength, as discussed more below. We conclude that the addition of manual therapy with rest immediately following the cessation of the activities at fault is beneficial for symptoms and tissue pathologies that develop due to repetitive work performance.

Neuropathies such as carpal tunnel syndrome are very common in work related disorders. In this model, we have previously shown that nerves become progressively inflamed, evidenced by progressive myelin degeneration, macrophage recruitment, fibrosis, erratic neural discharge in the entire range of neuron types, and decreased NCVs (Elliott et al., 2009, 2010; Bove et al., 2019; Barbe et al., 2021). Here we report a NCV reduction of 27%, which is comparable to declines in a study examining nonhuman primates (Macaca fascicularis), where voluntary repetitive pinching for a prolonged period led to a 25–31% decline from baseline (Sommerich et al., 2007). In that study, MRIs showed enlargement and heightened signal intensity of the affected nerves near the proximal end of the carpal tunnel at the time of the NCVs slowing, consistent with nerve inflammation. These match our current and past findings that nerve inflammation is a key contributor to NCVs declines and electrophysiological changes (Bove et al., 2016, 2019; Govea et al., 2017), although our correlation results suggests that muscle inflammation may also contribute to declines in NCV.

Grip strength declined 29% in the TASK rats from their pre-task levels, and were 68% lower than age-matched non-task rats by study end (Bove et al., 2019; Barbe et al., 2020a, 2021). There are many possible contributors to grip strength, including median nerve pathology, muscle inflammation, and fibrosis (Stauber et al., 1996; Schafers et al., 2003; Stauber, 2004; Beyreuther et al., 2007; Barbe et al., 2020b, 2021). The correlational results in this study suggest that both nerve and muscle inflammation can be implicated in grip strength declines (moderate and negative correlational findings between grip strength and nerve CD68/ED1^+^ numbers and muscle TNFα levels). IL-10 is a potent anti-inflammatory cytokine (Iyer and Cheng, 2012). The higher levels of muscle IL-10 seen in MTR rats, compared to REST rats, may be the reason why their grip strength responses were similar to age-matched non-task rats (FRC rats), while grip strength remained slightly lower than normal in REST rats.

Although we have shown here that manual therapy offered a few advantages compared to rest alone, it must be pointed out that we have previously shown in the same model that manual therapy provided concurrent with the task performance effectively prevented the development of behavioral, electrophysiological, and tissue pathologies (Bove et al., 2016, 2019; Barbe et al., 2021). In contrast to the delayed rest and manual therapy approach used in this current study, with preventive approach task-induced behavioral declines never occurred, and performance even improved (Bove et al., 2016). Others have published similar results using other force-based methods. Using a method designed to emulate cross-fiber massage, treatment augmented pain induced by experimental subcutaneous inflammation (Loghmani et al., 2021). A robot-assisted mechanical therapy resembling deep tissue therapy was shown to prevent behavioral and histological markers of muscle damage following experimental stroke (Sen et al., 2017). In studies using a cyclic loading roller, muscles that underwent immediate treatment after highly intense exercise (continued every 24h for 4days) exhibited only minimal disruption of myofibers and lower inflammatory cell infiltration than muscles that received the same treatment 48h later (Haas et al., 2013). These findings combined with ours suggest that preventive manual therapy treatments are preferable over attempts to reverse developed pathologies. Consideration must also be made for recurrence. Although we did not study recurrence here, since our data show that some rats responded better than others to the reversal treatment (as shown in the scatter plots); it follows that these rats would seem more likely to develop their symptoms should they return to work. Unpublished findings from the lab in which a number of rats that returned to work after a 4-week rest quickly developed tissue inflammation and indices of discomfort, in support of this recurrence hypothesis. This may parallel the situation in humans who return to work after symptomatic recovery only to suffer recurrence (Ruseckaite and Collie, 2013; Oranye, 2017). This also supports the role of prevention, in terms of human suffering and lost work.

As we have reported previously (Bove et al., 2016, 2019; Barbe et al., 2021) that the manual therapy treatment used here when performed on awake rats was well tolerated. In this study, rats initially tended to pull away from the treatment (Figure 1), a behavior rarely seen in previous studies. We interpret this finding as a measure of tenderness. Since there was no behavior requiring higher level functioning, such as struggling or biting, it is likely that this was a spinal reflexive behavior. This tendency to withdraw resolved gradually over the course of the experiment, consistent with the resolution of tenderness. When this type of treatment is provided to humans, their responses, including withdrawals and facial expressions, are key to guiding the provider to use the appropriate force. Indeed, providers typically ask patients to tell them if pressures and locations are painful as a guide not only to the appropriate use of pressures, but also to gather diagnostic information as the treatment is given. Our current data support that the use of hands rather than instruments on unanesthetized animals allows for necessary feedback to help guide the treatment, which needs to be continuously modified.

Two limitations of this study need to be considered. First, only female rats were included. The force transducer sensitivity of our model setup is currently tailored to the pulling strength of female rats, so inclusion of males may have reduced data quality and made the interpretation of findings more difficult and would have added sex as a potential confounder. Another reason for our focus on one sex is that human females have a higher incidence of work-related musculoskeletal disorders than males (Gerr et al., 2002; Cote, 2012). However, since human males also develop these disorders (Sharma and Singh, 2014), future studies are encouraged to include males. Additionally, this is an animal study rather than a clinical trial using humans. That said a key strength of our model is that it is an operant model in which rats develop changes in the same manner as humans involved in prolonged work-related upper extremity tasks (Barr and Barbe, 2002). We focused on neuromuscular changes only in this current study. Other tissues of the forelimb and forepaw also respond to the overuse injury task, including bone and tendon, and a separate study is underway to examine their response to the task and treatments.

## Conclusion

Manual therapy performed for 7weeks in conjunction with rest improved many but not all of the behavioral and tissue attributes assayed in rats with multiple pathologies developing from 14weeks of a repetitive work task. The 7-week rest also improved several of the functional and neuromuscular pathologies, although the manual therapy combined with rest ameliorated cold sensitivity and elevated muscle IL-10 levels more than rest alone. These results can be compared to results in this same model where the treatment was performed concurrently with the task, and where the pathologies do not develop (Bove et al., 2016, 2019; Barbe et al., 2021). Taken as a whole, the data support that prevention of pathologies is desirable in repetitive tasks that are known to lead to musculoskeletal disorders.

## Data Availability Statement

The raw data supporting the conclusions of this article will be made available by the authors, without undue reservation.

## Ethics Statement

The animal study was reviewed and approved by Temple University Institutional Animal Care and Use Committee.

## Author Contributions

MB and GB were responsible for the research design. MB, SP, MH, GC, MA, JD, GC, and GB, each, contributed substantially to various aspects of the data acquisition, analysis, and interpretation. MB, SP, and GB drafted the paper and substantially revised it. All authors contributed to the article and approved the submitted version.

## Funding

Research reported in this publication was supported by the National Center of Complementary and Integrative Health of the National Institutes of Health under Award Number AT009350 to MB and GB. The content is solely the responsibility of the authors and does not necessarily represent the official views of the National Institutes of Health.

## Conflict of Interest

The authors declare that the research was conducted in the absence of any commercial or financial relationships that could be construed as a potential conflict of interest.

## Publisher’s Note

All claims expressed in this article are solely those of the authors and do not necessarily represent those of their affiliated organizations, or those of the publisher, the editors and the reviewers. Any product that may be evaluated in this article, or claim that may be made by its manufacturer, is not guaranteed or endorsed by the publisher.
